# 3D metamaterial ultra-wideband absorber for curved surface

**DOI:** 10.1038/s41598-023-28021-4

**Published:** 2023-01-19

**Authors:** Mahdi Norouzi, Saughar Jarchi, Mohsen Ghaffari-Miab, Meisam Esfandiari, Ali Lalbakhsh, Slawomir Koziel, Sam Reisenfeld, Gholamhosein Moloudian

**Affiliations:** 1 Faculty of Technical and Engineering, Imam Khomeini International University, Qazvin, Iran; 2 Faculty of Electrical and Computer Engineering, Tarbiat Modares University, Tehran, Iran; 3 School of Engineering, Macquarie University, Sydney, Australia; 4 School of Electrical and Data Engineering, University of Technology Sydney (UTS), Sydney, NSW Australia; 5 Department of Engineering, Reykjavik University, 102 Reykjavik, Iceland; 6 Faculty of Electronics, Telecommunications and Informatics, Gdansk University of Technology, 80-233 Gdansk, Poland; 7 Tyndall National Institute, University College Cork, Cork, T12R5CP Ireland

**Keywords:** Electrical and electronic engineering, Optical physics

## Abstract

This paper proposes a three-dimensional metamaterial absorber based on a resistive film patch array to develop a low-cost, lightweight absorber for curved surfaces. An excellent absorption over a large frequency band is achieved through two different yet controllable mechanisms; in the first mechanism, a considerable attenuation in the wave power is achieved via graphite resistive films. The absorption is then intensified through magnetic dipoles created by the surface currents, leading to absorption peaks. The simulation results of the absorber show that a broadband absorption greater than 85% is achieved over 35–400 GHz for both TE and TM polarization waves at normal incidence. The structure has more than 167% and 80% absorption bandwidth above 85% and 90%, respectively. It is shown that the proposed metamaterial absorber is independent of incident wave polarization. In addition, the structure is insensitive to incident angles up to 60° for TE mode and full range angle 90° for TM mode. To describe the physical mechanism of the absorber, E-field, power loss density and surface current distributions on the structure are calculated and shown. Moreover, the oblique incidence absorption efficiency is also explained. This absorber paves the way for practical applications, such as sensing, imaging and stealth technology. In addition, the proposed structure can be extended to terahertz, infrared and optical regions.

## Introduction

The advent of materials with a negative refraction index has led to extensive research in the area of metamaterials over the past decade. Metamaterials are attractive not only for their marvelous electromagnetic properties but also for their diverse current and emerging applications^1^, such as transmitters^2^, sensors^3^, spatial modulators^4^, IR camouflage^5^, thermophotovoltaic^6^, phase modulator^7,8^, filter^9^ and wireless communication^10^ and absorbers^11^. The idea of a substance that absorbs all the radiation waves, regardless of the frequency or incident angle, was introduced a century ago by Planck's law^12^. In order to have a practical metamaterial absorber on uneven structures, the absorber should be independent of the incident wave polarization and angle^13^. In general, reduction of sensitivity to electromagnetic wave polarization can be easily achieved by using the symmetric design of metallic arrays on the surface of the absorber^14^.

Conventionally, absorbers have a sandwich structure with multiple layers where absorption is a function of the incident angle for both TE and TM modes^15,16^. Some studies have shown that increasing the angle of incidence negatively affects the impedance matching, resulting in higher reflection, leading to a lower absorption rate^17,18^. As a result, designing a low-cost polarization-insensitive absorber capable of efficiently operating under large angles of incidence has been a challenge. Another recent approach for designing absorbers is based on artificial intelligence (AI) algorithms, as described in Ref.^19^. As can be found in the literature, such AI-based approaches can be built and improved using nature-based algorithms such as particle swarm optimization^20,21^, different versions of artificial neural networks^22–31^, ant colony^32,33^, gray golf optimization^34,35^, genetic algorithm^36^, or other multi-objective optimization algorithms^37–40^.

This paper presents a polarization-insensitive absorber composed of resistive films along with a conductive backplate. In this design, resistive metamaterial films are used to create a lossy environment to achieve a broadband response with high absorption up to 60° and 90° for TE and TM mode radiation angles, respectively. Furthermore, the proposed absorber is a suitable candidate for the curvatures thanks to its stable performance for large incident angles.

## Design and simulation

**Figure 1:**
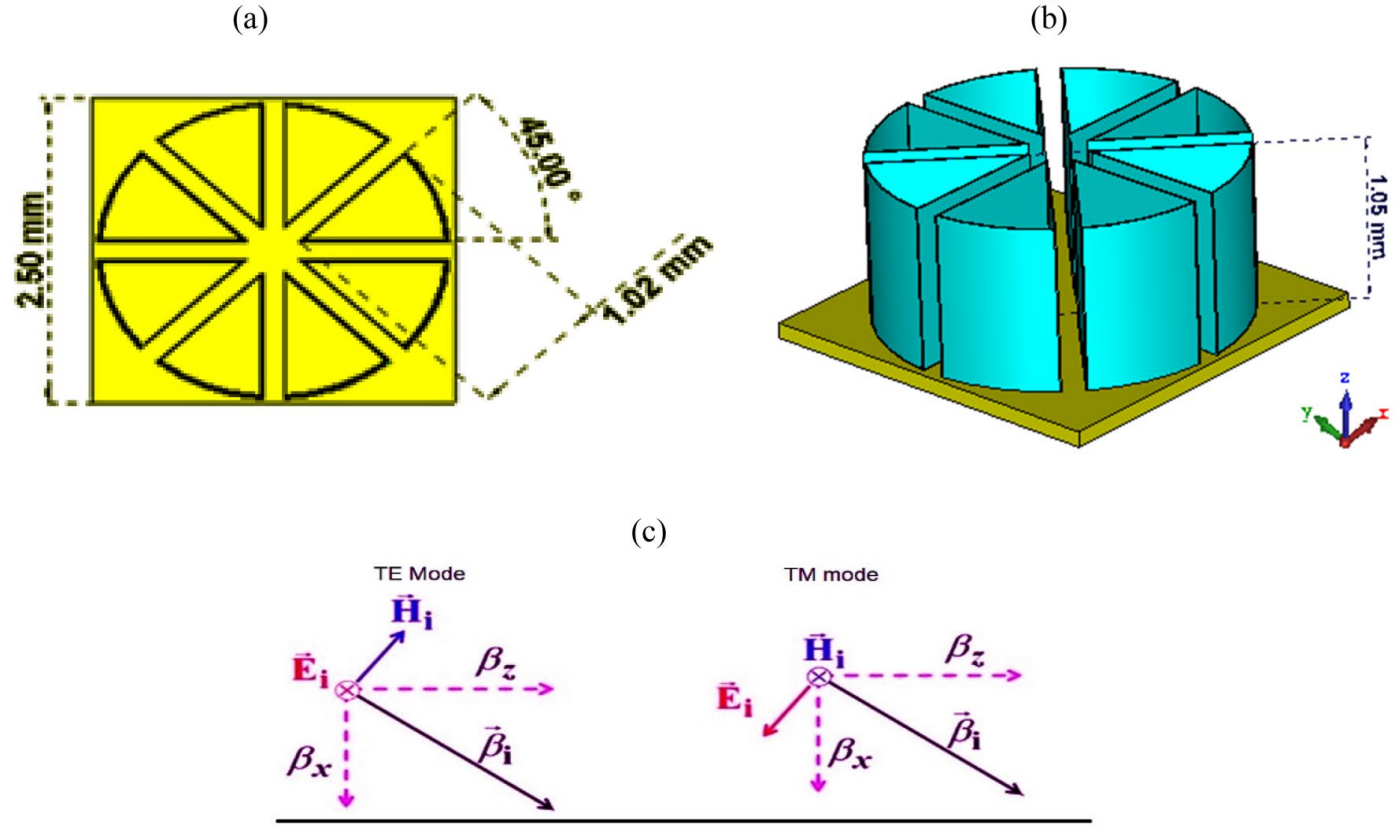
Absorber structure (**a**) top view and (**b**) 3-D view (**c**) schematic view of TE and TM Mode.

Figure 1a,b show the configuration of the proposed absorber consisting of eight 45-degree hollow sections made of graphite, with a thickness of 0.01 mm, a radius of 1.02 mm and a spacing of 0.17 mm, placed on a copper plate (thickness = 0.1 mm). The electric conductivity of graphite is 1000 [S/m], and the electric conductivity of copper is 5.8e + 007 [S/m]. The pizza shape is chosen for this structure because the horizontal films absorb, and other lossy films absorb waves at different angles. The schematic view of TE and TM mode is shown in Fig. 1c.

The structure has central symmetry and is expected to be non-sensitive to the input wave polarization. Over the past decade, several computational approaches have been successfully applied to optics and electromagnetic problems, such as finite element^41–49^, finite difference time domain^50–65^, and finite‐difference frequency domain^66–72^. In this work, the results are predicted using CST Microwave Studio Transient solver, which is based on the Finite Integration Technique (FIT). In simulations, the unit cell is located in the x–y plane and the wave radiates upon it in the z-direction. In this simulation, we use periodic boundary conditions^73^.

## Result and discussion

**Figure 2:**
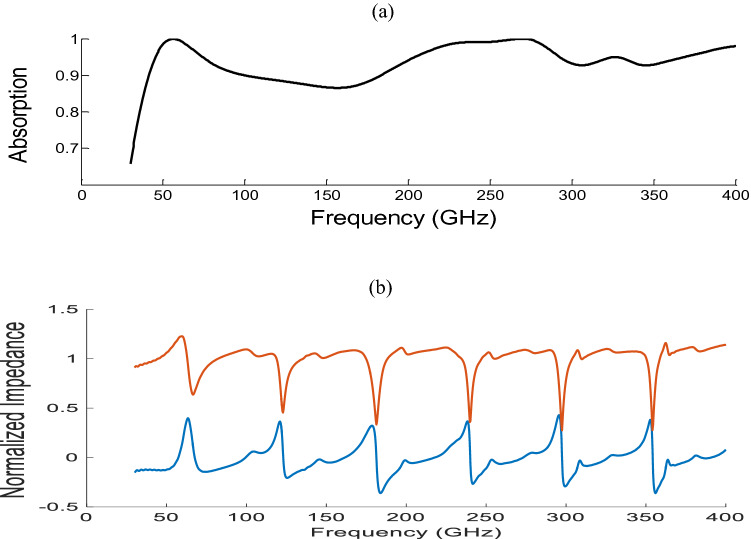
Absorption of the MA with (**a**) the normal incidence and (**b**) normalized impedance of the proposed absorber.

The obtained results for the amplitude of absorption coefficients in the frequency range of 35–400 GHz are shown in Fig. 2a. At the resonance frequencies of 57 GHz and 270 GHz, the absorption rate is 99%; however, it is above 85% for 35–400 GHz bandwidth, following the absorption rate determined in Ref.^74^. An EM wave incident onto the surface is reflected and transmitted. Minimizing the reflection coefficient R(*W*) and the transmission coefficient T(*W*) results in high absorptivity. The amount of absorption A(*W*) is considered as^75^:

1 $$A(W) = 1 - R(W) - T(W).$$

The amount pf transmission is almost zero because the metal ground and reflection coefficient is calculated by:

$$R(W) = \frac{{Z(W) - Z_{0} (W)}}{{Z(W) + Z_{0} (W)}},$$

where Z(*W*) and $Z_{0} (W)$ are the impedances of the metamaterial unitcell absorber and free space, respectively. Therefore, the zero-reflection condition is satisfied when $Z(W) = Z_{0} (W)$.

The impedance of the proposed metamaterial unitcell is normalized with the impedance of free space in a normal incident, shown in Fig. 2b. As can be seen, when the real part of the impedance is close to unity and the imaginary parts are zero, the amount of absorption becomes high. This is inevitable that the zero-reflection condition differs under TE and TE modes. For instance, at oblique incidence, the reflection coefficients for the TE and TM polarizations are given by:

2 $$R_{TE} = \frac{{Z(W)COS\theta_{i} - z_{0} COS\theta_{t} }}{{Z(W)COS\theta_{i} + z_{0} COS\theta_{t} }},$$

and

3 $$R_{TM} = \frac{{Z(W)COS\theta_{t} - Z_{0} COS\theta_{i} }}{{Z(W)COS\theta_{t} + Z_{0} COS\theta_{i} }},$$

where $\theta_{i}$ and $\theta_{t}$ are the incident and transmission angles, respectively. In this situation, the amount of absorption changes when the incident angles are varied.

**Figure 3:**
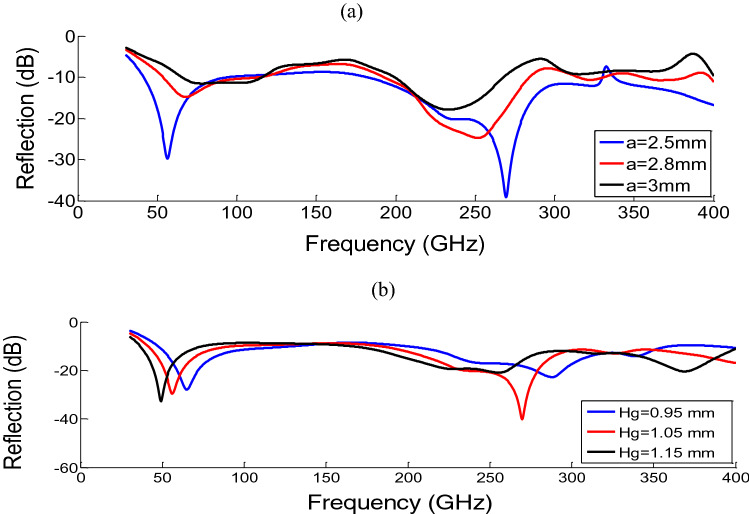
Reflection of the MA with the normal incidence for the physical parameters of (**a**) a (width of the unitcell), Hg = 1.05 and (**b**) Hg (height of the graphite film), a = 2.5.

The reflection curve is investigated for two parameters of width of the unitcell (a) and height of the graphite film (Hg) shown in Fig. 3a,b.

**Figure 4:**
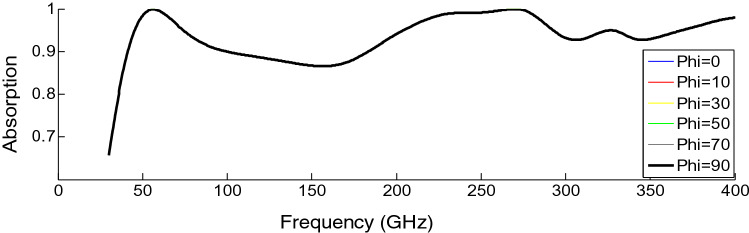
Absorption curves as a function of polarization angle (phi) under the normal incidence with a = 2.5 mm and Hg = 1.05 mm.

The absorption of the structure for various polarization angles is investigated and demonstrated in Fig. 4. Due to the structure symmetry, the absorber exhibits non-sensitivity to the polarization of the incoming waves. Furthermore, the absorber has nearly zero transmission due to its copper back layer, whose thickness is greater than the skin depth of the incident wave at the entire frequency band.

### Absorption mechanism in the normal incident

**Figure 5:**
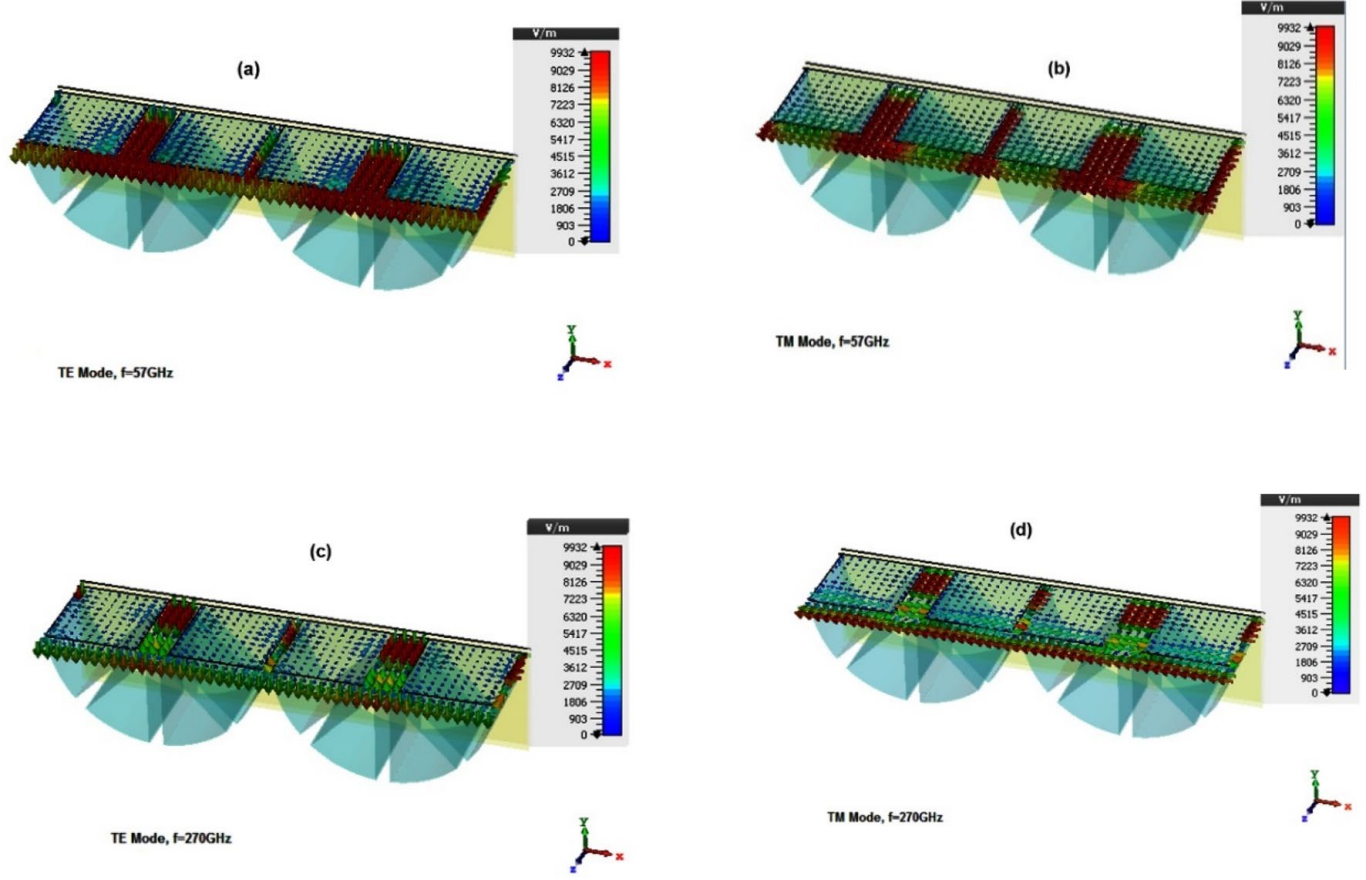
The E-field distributions at (**a**) f = 57 GHz, TE mode (**b**) f = 57 GHz, TM mode (**c**) f = 270 GHz, TE mode and (**d**) f = 270 GHz, TE.

In order to visualize the absorption mechanism at resonance frequencies, the electrical field distribution for two modes of TE (Ey) and TM (Hy) is shown in Fig. 5. The figure indicates that the electrical field is more concentrated on resistive film edges. This is because both plates form a capacitor and the electric field is higher at the edges of the capacitor.

**Figure 6:**
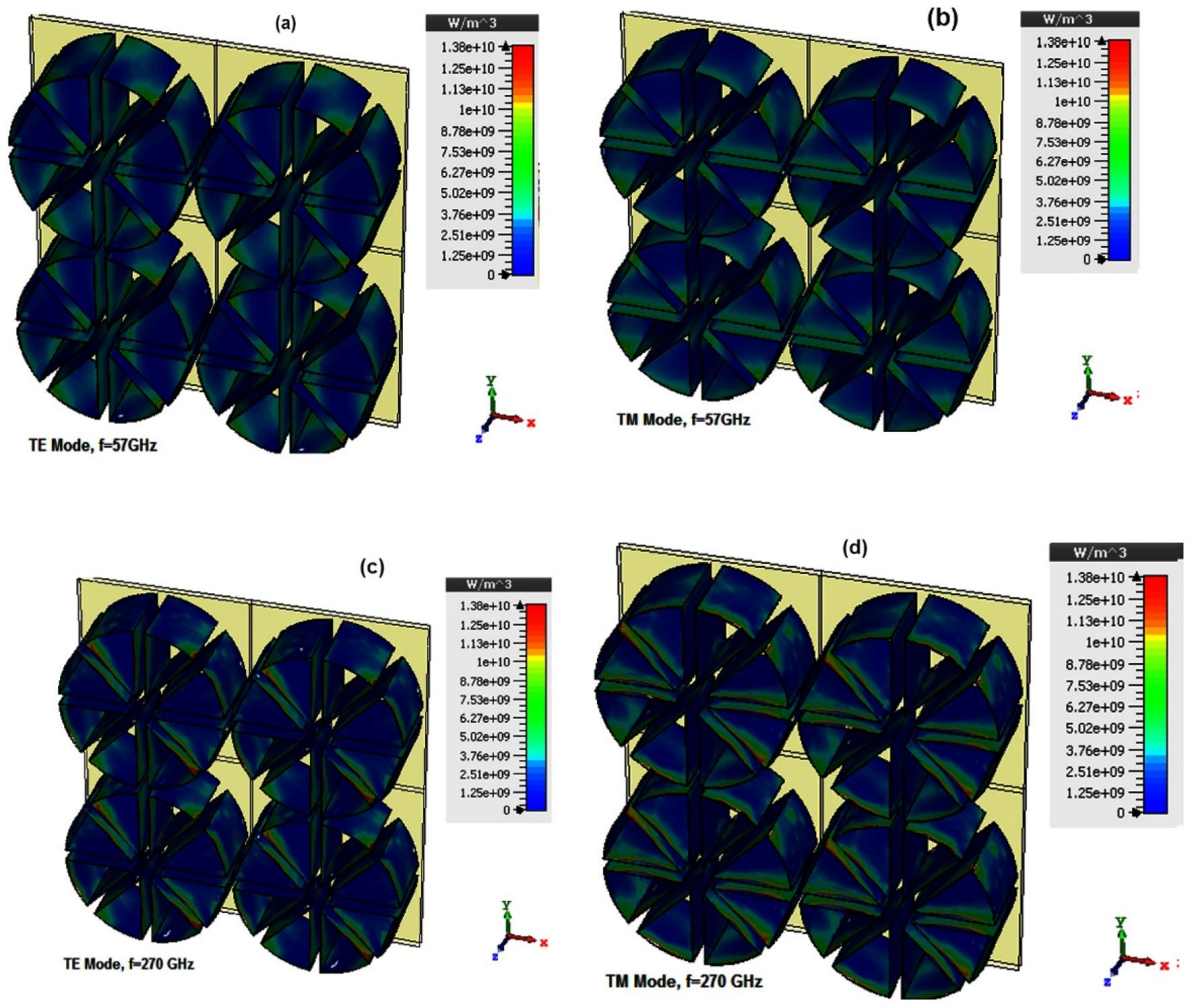
The power loss density at (**a**) f = 57 GHz, TE mode (**b**) f = 57 GHz, TM mode (**c**) f = 270 GHz, TE mode and (**d**) f = 270 GHz, TE mode.

The power loss density is depicted in Fig. 6. As expected, resistive plates perpendicular to the E direction have a negligible contribution to the power dissipation of the incoming waves. Indeed, the dissipation ratio of the input wave in resistive films depends on their orientation to the direction of the electric field.

**Figure 7:**
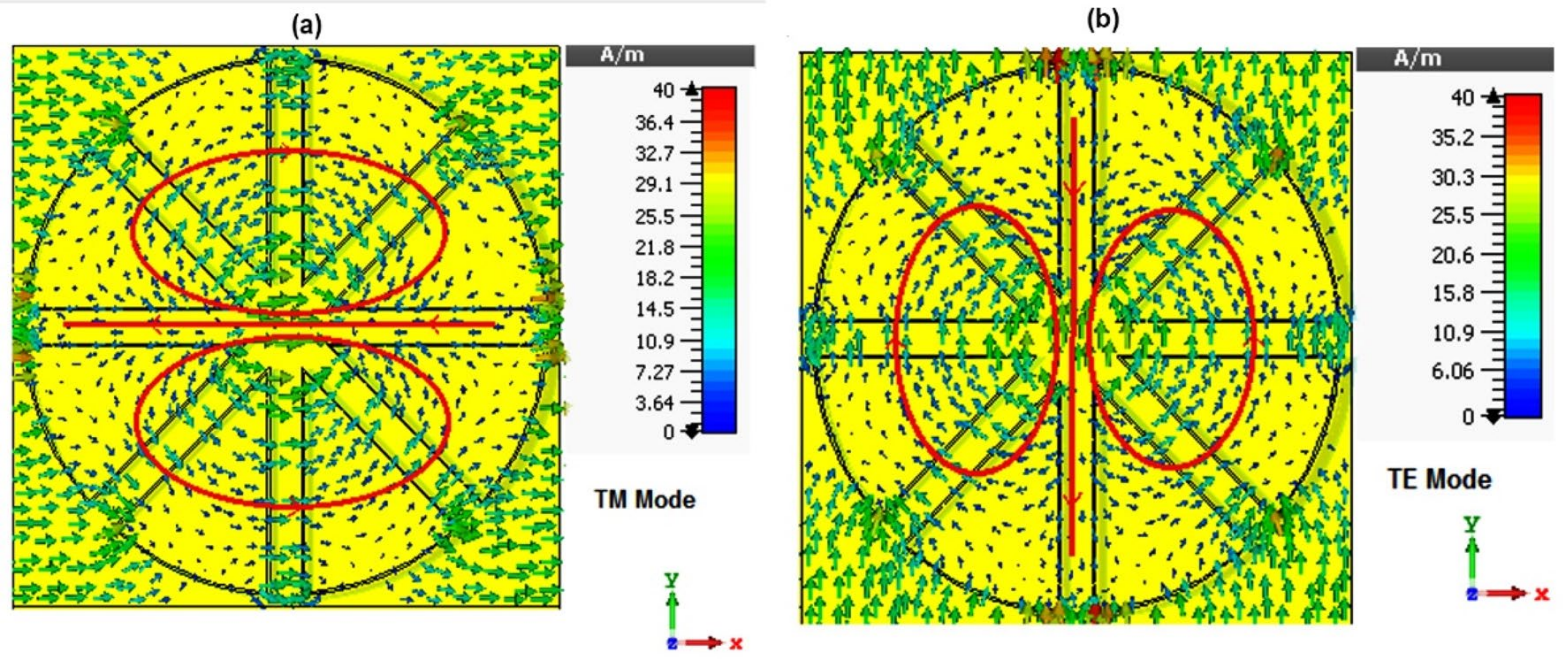
The surface current distribution at the central frequency (220 GHz) for (**a**) TM and (**b**) TE mode.

Figure 7 provides the absorber surface current distribution at the central frequency of the operating frequency band, giving a better insight into the high absorption of the structure. As shown in this figure, eight parallel current groups are formed along the edges of the film. A magnetic dipole is formed by these eight current loops, creating a magnetic response strongly coupled to E and H input fields, creating high-absorption peaks and ultimately aggravating absorption^76,77^. In conjunction with the primary absorption by the resistive nature of the graphite films, this phenomenon completes the process of absorbing electromagnetic waves in this absorber. To further improve the results, surrogate based optimizations can be as previously used in other electromagnetic devices^78–80^.

### Absorption in TE and TM modes under oblique incident wave

**Figure 8:**
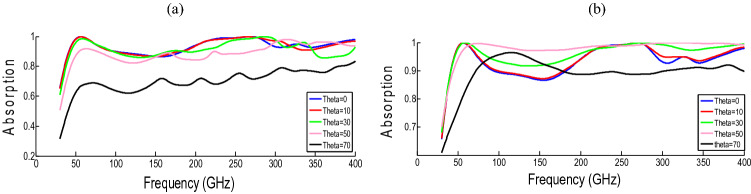
The absorption is based on oblique incidence (theta) (**a**) TE mode (**b**) TM mode.

Figure 8a,b show the absorption diagrams under oblique incidents with different radiation angles (θ). As the incident angle increases, the resonance points are displaced. In detail, the operating frequency band shrinks as the incident angle increases, but absorption remains above 75% for TE mode and is insensitive to incident angle up to 60°. The proposed absorber exhibits more resistance to large incident angles in the case of TM mode, showing a negligible frequency shift.

### Absorption difference mechanism between TE and TM modes

**Figure 9:**
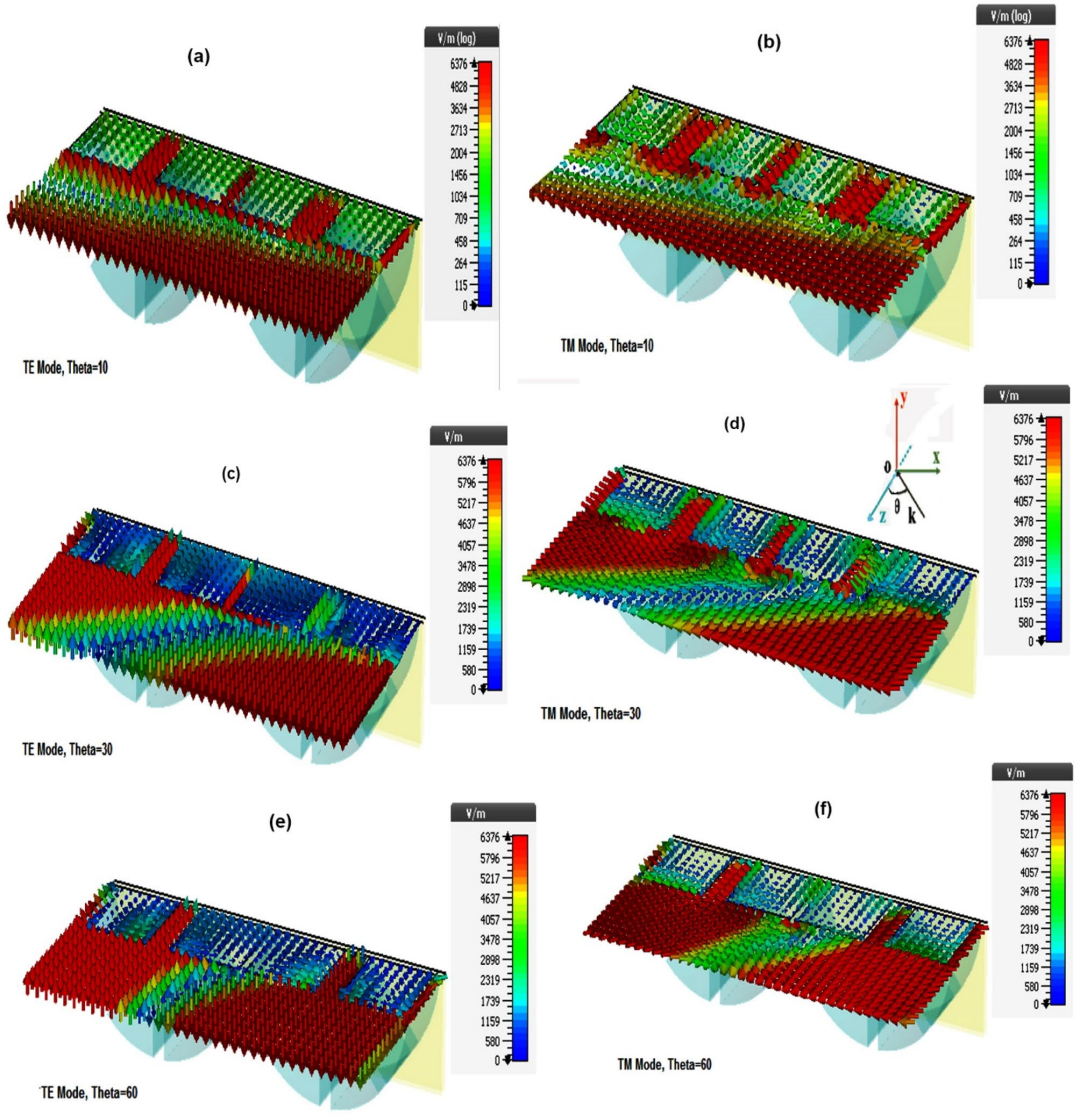
E-field distributions of different oblique incidence at the central frequency (220 GHz) for (**a**) Theta = 10, TE Mode (**b**) Theta = 10, TM Mode (**c**) Theta = 30, TE Mode (**d**) Theta = 30, TM Mode (**e**) Theta = 60, TE Mode (**f**) Theta = 60, TM Mode.

**Figure 10:**
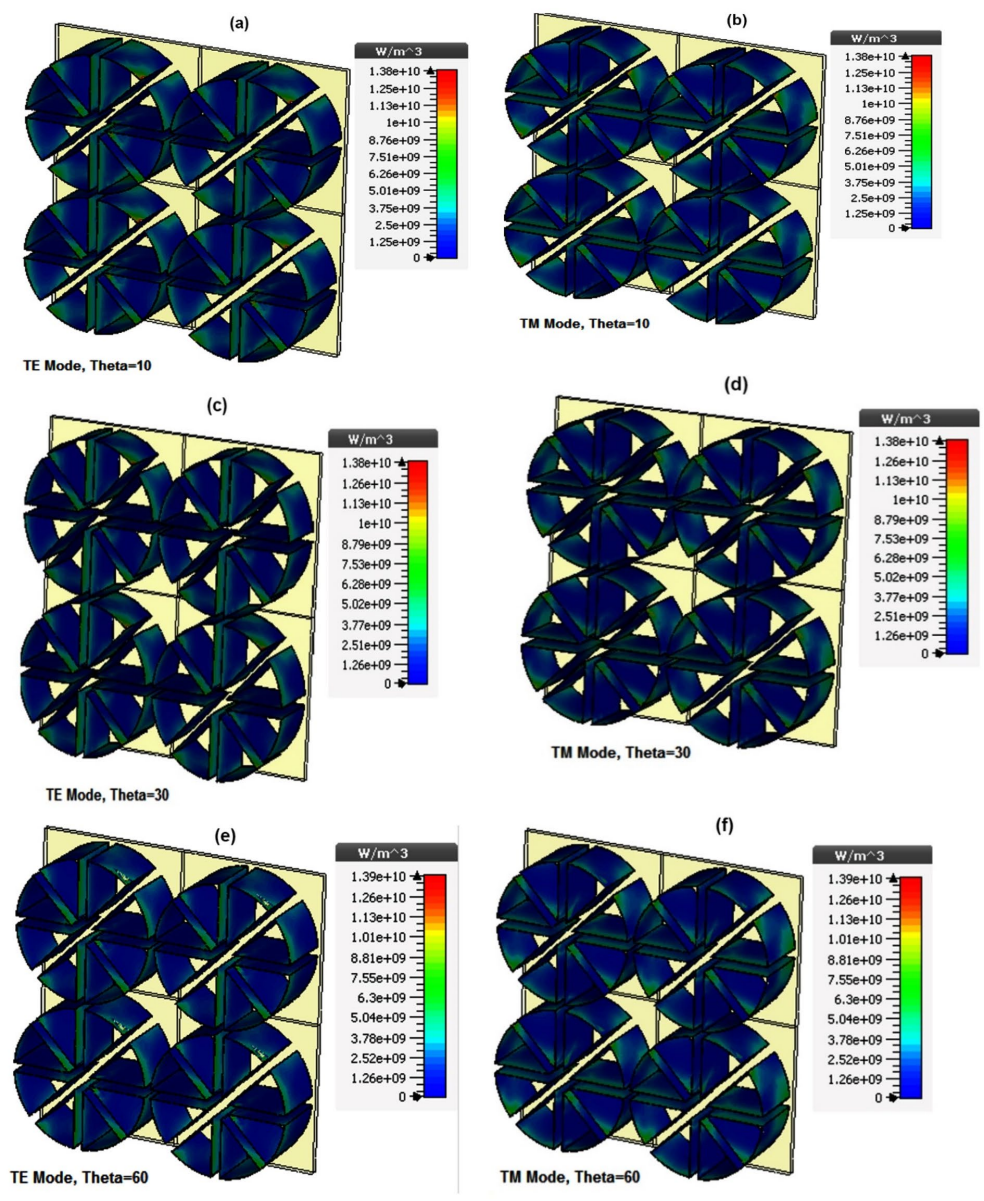
Power loss density based on oblique incidence at the central frequency (220 GHz) for (**a**) Theta = 10, TE Mode (**b**) Theta = 10, TM Mode (**c**) Theta = 30, TE Mode (**d**) Theta = 30, TM Mode (**e**) Theta = 60, TE Mode (**f**) Theta = 60, TM Mode.

The electric field distribution and power loss density for the incident angles of 10°, 30° and 60° at 220 GHz are shown in Figs. 9 and 10, respectively, highlighting the differences between the input power dissipation in TE and TM modes.

As mentioned, the electric field propagates perpendicular to the resistance films; however, increasing the angle in TM mode makes the electric field component more tangent to the films, leading to more losses. For example, the electric field component at 30° is much more tangent to resistance films than at an angle of 10° (Fig. 9).

In addition, increasing the incident angle for TM mode changes the electric field direction from the single component in the x-direction to two components of x and z. Therefore, unlike the TE mode, the absorption in the TM mode increases as the incident angle increases.

As shown in Fig. 10, for the TE mode (where the electric field remains at all angles in the y-direction), horizontal resistive films (in the x-direction) do not affect the power dissipation because they are aligned perpendicular to the E field.

### The effect of absorber on the radar cross section (RCS) of a curved structure

**Figure 11:**
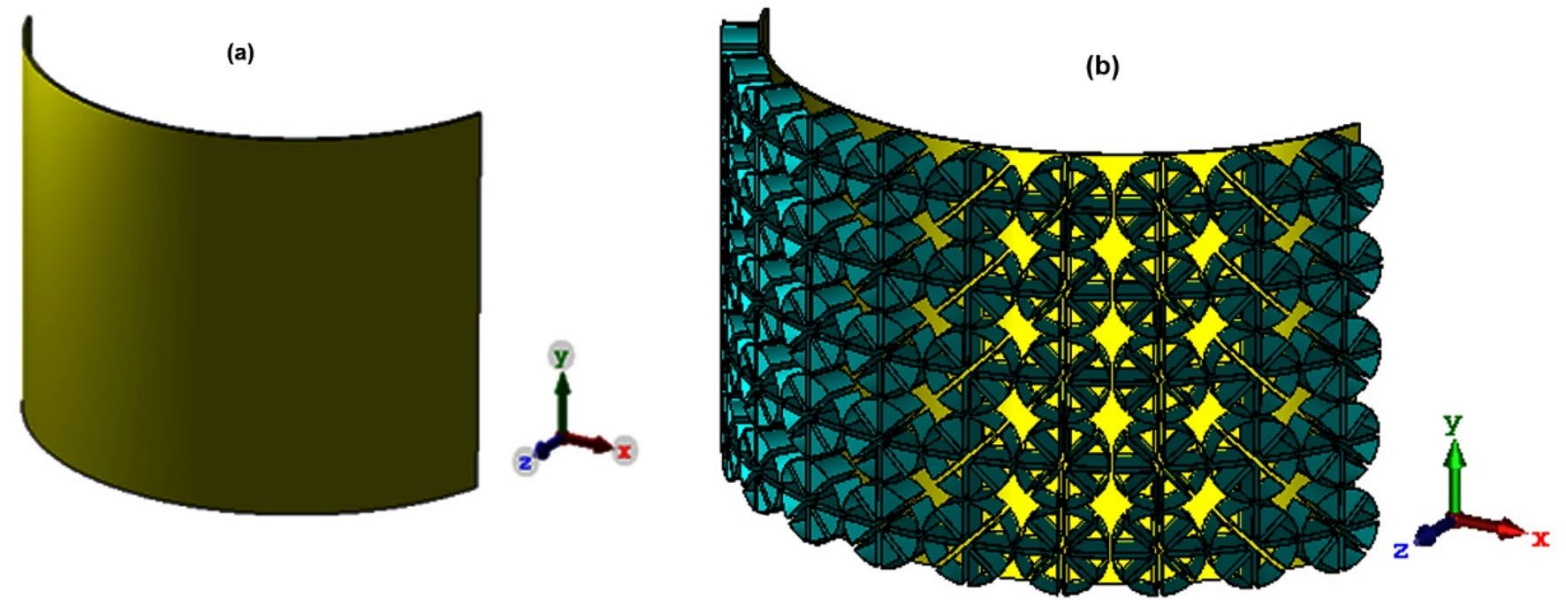
Curved structure (**a**) without and (**b**) with the proposed absorber.

In order to validate the absorber performance in reducing radar cross-section, a curved copper surface is used as shown in Fig. 11a,b. After radiating the electromagnetic wave to the plate, the RCS value is measured. Finally, the absorber coating is fixed on the surface and the RCS measured again and compared with its value in the previous state.

**Figure 12:**
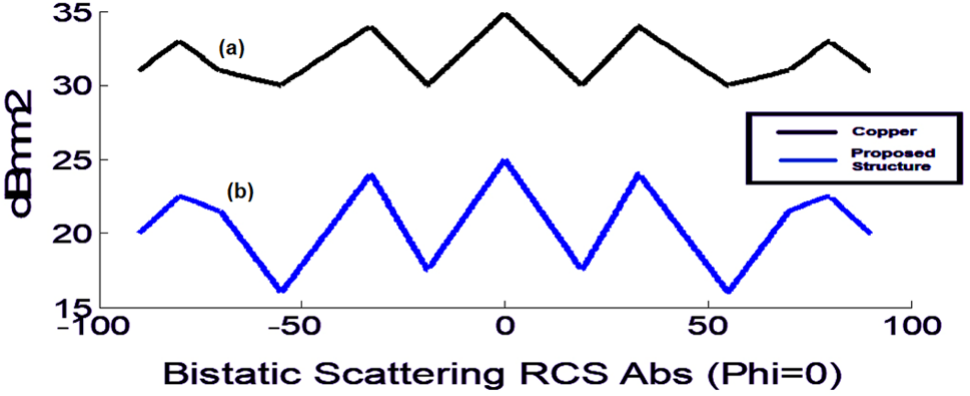
Curved structure (**a**) without and (**b**) with the proposed absorber.

For this purpose, a copper semi-cylinder with a radius of 10 cm, the electrical conductivity of 8.57 × 10 s/m and a thickness of 0.18 mm was created (Fig. 11a), and a plane wave with the open boundary conditions was radiated to it from the front in the CST software environment. After analyzing the structure, the RCS is obtained at different angles (θ) at the central frequency when the polarization angle is zero. Once again, the surface is covered by the proposed absorber (Fig. 11b), and the RCS results for the two cases are shown in Fig. 12. The comparison between the two graphs shows the RCS is considerably smaller with the absorber, and this RCS reduction is visible at all angles. The RCS comparison diagram shows a difference of as large as 10 dB between the two modes. In fact, when the proposed metamaterial absorber coating covers the curve surface, it is hidden from the radar view and has a 90% reduction in RCS compared to a bare metal plate. Table 1 compares the proposed absorber with some of the recently published works. As can be seen from this table, the proposed absorber is superior to previous structures in terms of bandwidth and sensitivity to the incident wave angle. In addition, it is worth mentioning that the proposed absorber is lightweight, thanks to its graphite-based configuration.

**Table 1:** Comparison of proposed absorber with previous works.

References	BW (%)	Frequency band (GHz)	Unit cell volume (λ × λ × λ)	Maximum incident angle with greater 85% absorption		Polarization sensitivity (yes/no)	Configuration
				TE mode	TM mode		
^81^	44	58.6–91.4	0.64 × 0.64 × 0.64	30	90	No	U shaped resistive film
^82^	100	3.9–12	0.32 × 0.32 × 0.12	45	45	No	Carbon-loaded acrylonitrile butadiene styrene (ABS) polymer
^83^	150	5.1–40	0.5 × 0.5 × 0.17	55	70	No	Flaky carbonyl iron and polyther
Proposed structure	167	35–400	0.33 × 0.33 × 0.14	60	90	No	Graphite film

## Conclusion

This paper proposes a straightforward yet highly efficient absorber based on graphite resonators that require neither lumped elements nor dielectric substrates, contributing to affordable manufacturing. Furthermore, due to its symmetric geometry, the proposed absorber demonstrates a stable response over a large frequency bandwidth of 167%, regardless of the incoming wave polarization. The absorber is flexible and suitable for curved surfaces, showing acceptable performance under oblique incidents up to 60° and 90° for TE and TM modes, respectively.

## Data Availability

The datasets used and/or analyzed during the current study are available from the corresponding author upon reasonable request.
